# Fc Gamma Receptors and Their Role in Antigen Uptake, Presentation, and T Cell Activation

**DOI:** 10.3389/fimmu.2020.01393

**Published:** 2020-07-03

**Authors:** Fabian Junker, John Gordon, Omar Qureshi

**Affiliations:** ^1^Roche Pharma Research and Early Development, Pharmaceutical Sciences, Roche Innovation Center Basel, F. Hoffmann-La Roche Ltd, Basel, Switzerland; ^2^Celentyx Ltd, Birmingham Research Park, Birmingham, United Kingdom

**Keywords:** FcγRs, Fc gamma receptors, antigen presentation, immune complexes, T cell activation, autoimmunity

## Abstract

The cellular uptake, intracellular processing, and presentation of foreign antigen are crucial processes for eliciting an effective adaptive host response to the majority of pathogens. The effective recognition of antigen by T cells requires that it is first processed and then presented on MHC molecules that are expressed on other cells. A critical step leading to the presentation of antigen is delivering the foreign cargo to an intracellular compartment where the antigen can be processed and loaded onto MHC molecules. Fc-gamma receptors (FcγRs) recognize IgG-coated targets, such as opsonized pathogens or immune complexes (ICs). Cross-linking leads to internalization of the cargo with associated activation of down-stream signaling cascades. FcγRs vary in their affinity for IgG and intracellular trafficking, and therefore have an opportunity to regulate antigen presentation by controlling the shuttling and processing of their cargos. In this way, they critically influence physiological and pathophysiological adaptive immune cell functions. In this review, we will cover the contribution of FcγRs to antigen-presentation with a focus on the intracellular trafficking of IgG-ICs and the pathways that support this function. We will also discuss genetic evidence linking FcγR biology to immune cell activation and autoimmune processes as exemplified by systemic lupus erythematosus (SLE).

## Introduction

The immune response can be divided broadly into innate and adaptive immunity. Innate immune cells, such as dendritic cells, monocytes, NK cells, and neutrophils among others, mount early responses to pathogens through recognition of conserved non-host pattern antigens, such as bacterial lipopolysaccharide (LPS) or viral RNA (1, 2). The adaptive immune system allows a more focused response to antigen discriminating self from non-self in such a way as to limit collateral damage to host tissues yet having the capacity to recognize and evolve a response to a wider diversity of foreign antigens. T and B lymphocytes are key players in the adaptive immune response as a result of their ability to recognize foreign protein antigen through highly diverse specific receptors and are also capable of long lasting immune responses to the antigens they encounter (3, 4).

While B cells can recognize antigens in their unprocessed form, T cells require antigens to be processed and presented on the surface of another cell by major histocompatibility (MHC) class I or MHC class II molecules. MHC class I molecules are broadly expressed and present endogenous antigens to CD8 T cells. MHC class II molecules are expressed mostly on specialized antigen-presenting cells (APCs) and certain other cell types. These present exogenously-derived antigens to CD4 T cells. Finally, there is a route whereby exogenous antigens are presented by MHC class I molecules termed cross-presentation (5) in which the dendritic cell (DC) plays a crucial role (6, 7).

DCs can broadly be grouped into three subsets in both mouse and human (8, 9): Plasmacytoid DC (pDC), myeloid/conventional DC1 (cDC1), and myeloid/conventional DC2 (cDC2). The different DC subsets have varying antigen presenting capabilities, with cDC1 cells being described as a subset with a high intrinsic capacity to cross-present antigens *via* MHC class I to activate CD8^+^ T cells as well as promoting T helper type 1 (Th1) and natural killer responses (as discussed in detail in later sections). In addition, other DC subsets, including certain cDC2 subsets (10), can also be induced to cross-present antigen. Plasmacytoid DCs, however, are largely thought to be ineffective at antigen presentation and T cell activation, although this issue remains somewhat controversial (11).

Inducing antigen specific T cell responses *via* MHC:antigen peptide–T cell receptor (TCR) interactions is essential for mounting long-lasting, effective immunity. This makes the uptake, subsequent intracellular processing and presentation of antigen in APCs critical. In the case of soluble protein antigens, these are to a considerable extent controlled by Fc-gamma receptor (FcγR) function, the subject of the present review.

## Protein Antigens are Internalized, Proteolytically Processed, and Loaded Onto MHC Molecules Inside The Cell for Antigen Presentation

MHC molecules present antigen peptides of length ~9–10 amino acids (aa) in the case of MHC class I, or 11–30aa in the case of MHC class II, held within a binding groove in the MHC molecules (12). Thus, for the bulk of extracellular antigens, proteolytic processing inside the cell is required (13).

In a healthy cell, MHC class I protein builds complexes with constituent cytoplasm-derived “self” peptides (14). Virus infected cells or tumor cells containing neo-antigens can however present “non-self” peptides to the T cells of the adaptive immune system resulting in their activation and culminating with the death of the unwanted host cell (15–17). Cytoplasmic proteins are initially degraded by the proteasome (18), then loaded into the endoplasmic reticulum (ER) lumen *via* the transporter associated with antigen processing (TAP) (19), and then incorporated into the MHC class I protein complex by the chaperone tapasin (20).

Proteolysis of antigens for MHC class II presentation occurs within the endolysosomal system and involves proteases such as cathepsins which are active at the acidic pH of these intracellular compartments (21). The efficiency of antigen-presentation in different cell types is related in part to the proteolytic potential of these intracellular compartments with specialized APCs containing a less acidic pH and protease content within the endo-lysosomal, favoring the conservation of peptide epitopes that can be loaded onto MHC (21). The loading of these antigen-derived peptides onto MHC II requires HLA-DM to facilitate the process (22).

In the case of cross-presentation, two pathways have been described that enable MHC class I molecules to be loaded with exogenous antigen. Antigens contained in the endosomal compartment can be shuttled into the cytoplasm, where they are processed similarly to conventional cytosolic antigens relying on TAP and proteasome function (23). Alternatively, lysosomal proteases such as cathepsin S have been suggested to degrade exogenous antigens already in the acidic compartment (24), where peptides are then loaded to intra-endosomal MHC class I molecules. This latter cross-presentation process has been termed the “vacuolar pathway” and exists for example in certain viral or bacterial infections (25). Cross-presentation is believed to be essential for host immunity to viral infections occurring in parenchymal cells. While MHC class II molecules present peptides derived from extracellular antigens, cytoplasmic, and nuclear antigens can also gain access to MHC class II compartments (26). Entry of these antigens into the endolysosomal system for delivery to MHC II compartments can be facilitated by both Lamp-2a (27) and autophagy (28).

## The Role of FcγRs in Internalizing Antigens

### Overview of FcγRs and IgG Binding

FcγRs bind to the IgG molecule through its Fc (fragment, crystallizable) portion (29). In humans, three groups of FcγRs have been described across a variety of cell types: FcγRI, FcγRIIA/B, FcγRIIIA/B (30). These are expressed in differing combinations at the surface membrane of the various immune cells (31). In the case of FcγRI, these include macrophages, neutrophils, eosinophils and DCs. For FcγRIIA, cell types include macrophages, neutrophils, eosinophils, platelets, and Langerhans cells as well as conventional, but not plasmacytoid, DCs (32). FcγRIIIA is found on natural killer (NK) cells and macrophages, as reviewed elsewhere (33). The inhibitory Fc gamma receptor FcγRIIB is found on B cells, mast cells as well as macrophages, neutrophils, and eosinophils. Importantly, it is also expressed on cDCs (32). Flow cytometry experiments suggest that it is unlikely that human pDCs, in contrast to mouse pDCs where expression of the inhibitory receptor FcγRIIB was claimed (34), express any FcγRs (32, 35). Moreover, some studies on pDCs may have included contaminating cDC (8). FcγRIIIB, which can be considered a decoy receptor since it lacks association with downstream signaling molecules (as discussed at later stages of this article), is mainly expressed on neutrophils but may under certain conditions also be expressed on other immune cells like basophils (29, 31).

Cell-type specific expression of mouse FcγRs (the activating FcγRI, FcγRIII, FcγRIV, and the inhibitory FcγRIIB) has also been assessed. Conventional mouse DCs were found to express FcγRI, FcγRIII, FcγRIV, and FcγRIIB (36, 37), while pDCs exclusively expressed FcγRIIB (34). FcγRIV was confirmed to be expressed on monocytes, neutrophils, and macrophages by flow cytometry (38). In addition, the inhibitory receptor FcγRIIB is expressed on B cells as well as monocytes and macrophages; mouse FcγRIII was described on NK cells, neutrophils, monocytes, and macrophages (31). In addition to relative expression patterns, in a recent comprehensive study using mouse and human peripheral blood, Kerntke et al. quantified the number of cell surface FcγR on derived immune cells including potential APCs such as B cells and monocytes (39). The authors found that on human monocytes, more FcγRIIA molecules were expressed compared to FcγRIIB. Likewise, in C57BL/6 mice, the activating FcγRIV was the most abundant receptor on non-classical monocytes, whereas FcγRIII was expressed to a much lesser degree on these cells, and FcγRIIB had even less copies. Overall, these results indicate that on monocytes, under steady-state conditions, activating FcγRs are overabundantly expressed with respect to the inhibitory FcγRIIB.

Importantly, mouse monocytes expressed greater numbers of the inhibitory FcγRIIB compared to their human counterparts. The actual fold difference was ~2.5x (for non-classical monocytes) and ~4x (for classical monocytes). Such inter-species differences should be taken into account when interpreting FcγR subtype-specific knockout experiments as discussed in later sections of this review.

In summary, APCs in both mouse and human, express a wide array of activating (and inhibitory) FcγRs, highlighting a potential role in antigen presentation through interaction with IgG immune complexes. Consequently, antibodies of the IgG class provide the bridge between antigen and FcγRs. FcγRI (also termed CD64) has the greatest affinity for IgG molecules, while FcγRs IIA/B/C (CD32) and FcγRIIIA/B (CD16) have lower affinities for IgG (40). The subdivision of IgG Into 4 distinct isotypes or subclasses—IgG1, IgG2, IgG3, and IgG4—provides additional complexity to Fc binding interactions (41) with the precise affinity of binding dependent on the IgG isotype (40).

In a comprehensive study using selectively expressed human FcγRs, Bruhns et al. found that, as monomers, IgG1, and IgG4 exclusively bound to FcγRI, whereas monomeric IgG2 failed to bind any of the FcγRs. Monomeric IgG3 also bound to FcγRI and additionally to FcγRIIIA. Taken together, the observations indicate that FcγRI can be considered a high-affinity IgG receptor most likely permanently occupied by monomeric IgG *in vivo* (31). Bruhns et al. also studied the affinities of IgG:antigen ICs with FcγRs. At high concentrations (10 μg/ml), all IgG-ICs bound to all FcγRs including the inhibitory FcγRIIB, although the latter was found to have a lower affinity for IgG1, IgG2, and IgG3 than other FcγRs. In summary, these findings indicate that IgG-ICs are able to differentially engage an array of FcγRs and preferentially cross-link low affinity receptors FcγRIIA, IIIA, and IIB, which under serum conditions are considered the main effector FcγRs on immune cells. In addition to the different affinities of FcγRs for IgG isotypes, the IgG-IC size plays an important role in the binding to FcγRs (35, 42, 43). Using ICs of defined sizes, the authors demonstrated that for ICs composed of IgG2 and IgG4, larger ICs exhibit increased interaction with the low-affinity human FcγRs over smaller ICs (42).

### FcγR Directed Internalization of Exogenous Antigens

**Figure 1:**
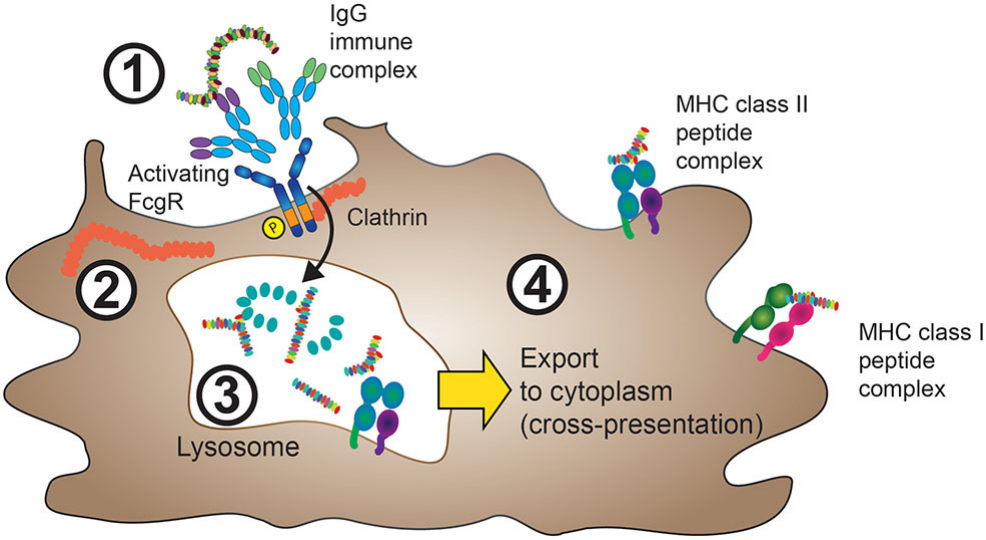
FcγRs mediate internalization of IgG-ICs. 1. IgG-ICs bind to low-affinity FcγRs at the cell surface of APCs. In the case of activating FcγRs, for instance FcγRIIA, these either contain intracellular ITAM sequences (purple), or are associated with ITAM containing FcRγ (not shown) as described in section The Molecular Machinery Downstream of FcγR and Figure 3. 2. Cross-linking of activating FcγRs leads to ITAM phosphorylation and clathrin-mediated internalization of the FcγR:IgG-IC aggregate with subsequent IgG-IC transport to lysosomes. 3. Inside the lysosomal compartment, both FcγRs and the internalized antigen are proteolytically degraded and eventually loaded onto MHC class II complexes, processes that depend on cathepsin, and HLA-DM activity (not shown). 4. MHC class II peptide complexes are shuttled from the endolysosomal compartment to the cell membrane in order to present antigen-derived peptides (usually 13–25aa in size) to cells of the adaptive immune system. Yellow arrow: In a process termed cross-presentation, non-“self” antigens internalized through FcγR cross-linking can also be shuttled to the cytoplasm, where they are processed similarly to endogenous proteins. The resulting 9–10-aa long peptides will then be incorporated in MHC class I protein complexes.

The loading of MHC class II molecules for foreign antigen presentation, as well as the loading of MHC class I molecules for cross-presentation, occurs inside the cell. Accordingly, foreign antigens need to be internalized before they can access the processing machinery, a process that is illustrated in Figure 1.

Opsonization (from the Greek “to make tasty”) of target antigens with IgG allows their detection by Fcγ receptors. The subsequent engagement of FcγRs by multivalent IgG-ICs leads to their clustering and initiation of signaling events that facilitate uptake of the antigen payload (44–47). In the case of phagocytosis of larger particles, actin-dependent cytoskeletal arrangements controlled by phosphatidylinositol 3-kinase, Cdc42, Rac1, and myosin motor proteins facilitate the uptake of the IgG-coated particle (48). Phagocytosis is an effective way to internalize cells as a source of antigens and allows subsequent antigen-presentation on both MHC class I and MHC class II molecules (44, 45). Smaller, soluble antigens bound to IgG in the form of ICs are delivered *via* FcγRs to lysosomes leading to degradation of both the ligand and the FcγR (49). Whilst monomeric IgG is usually shuttled between the extracellular space and the endosomal compartment *via* “recycling endosomes” upon internalization of the FcγR (50, 51), ICs and their associated antigen are processed intracellularly *via* a distinct route. Here, antigens are sorted to lysosomes for subsequent degradation and presentation (52) (Figure 1). Importantly, cross-linking of antigens is a key determinant for sorting antigen to the lysosomal compartment and preventing the recycling of an internalized IgG-IC (49, 53).

The uptake of soluble IgG-ICs utilizes some distinct molecular mechanisms when compared to those of phagocytosis. For example, Src kinases crucial for phagocytic uptake appear dispensable for the uptake of soluble IgG-ICs (54, 55). Again, in contrast to phagocytosis, the actin cytoskeleton appears not to be needed for the uptake of smaller ICs, whereas dynamin and clathrin have been found to play critical roles (56).

The Fc-mediated internalization of ICs is associated with subsequent enhancement in cellular antigen presentation efficiency (57). Expression of murine FcγRIII, transfected into FcγR-negative antigen-presenting B-lymphoma cells, conferred the ability to enhance the internalization of antigen-ICs in a manner dependent on tyrosine residues present within the cytoplasmic domain of the ITAM (immunoreceptor tyrosine-based activation motif)-containing FcR gamma chain (57). When these tyrosine residues were mutated, both the internalization of antigen and antigen presentation were impaired (a phenomenon that will be discussed in more detail in a later section). Of note, FcγRs appear capable of directing their cargo beyond the initial internalization step. The high affinity human IgG receptor FcγRIA was found to enhance the uptake of IgG-ICs in a manner independent of its C-terminus and FcR gamma chain (58). However, whereas ICs delivered by the full-length FcγRIA were directed primarily to MHC class II-containing compartments, those internalized by a truncated, tail-deleted FcγRIA were diverted to recycling compartments. The tail-deleted FcγRI-delivered ICs triggered inferior antigen presentation suggesting this intracellular routing of the IC may be important to the subsequent functional response (58).

FcγRs also play a critical role in antigen cross-presentation. Regnault et al. demonstrated in a mouse DC system that the internalization of IgG-ICs leads to an enhancement of cross-presentation compared to the fluid phase uptake of “naked,” non-IgG opsonized antigen (59). Importantly, FcγR engagement of the antigen-IgG complex was critical as such enhancement in presentation was not mimicked by the DC being exposed to an irrelevant IC in conjunction with the fluid phase uptake of the antigen. This finding suggested that FcγRs had a crucial role in directing the antigen to an intracellular compartment for subsequent processing and loading onto MHC class I molecules, a concept that will be discussed in detail in the following section.

The enhancement of cross-presentation also has the potential to enhance anti-tumor immunity. Dhodapkar et al. demonstrated that when DCs internalized tumor cells coated with an anti-tumor IgG antibody (against syndecan 1), they were able to trigger a superior expansion of antigen specific-T cells compared to DCs that had internalized tumor cells alone (60). Importantly those T cells were also superior at killing the tumor cell targets. Blockade of FcγRs using antibodies against FcγRI and FcγRII inhibited the generation of antigen-specific T cells further confirming the importance of FcγRs in this process.

In murine DCs, the inhibitory FcγR, FcγRIIB, is capable of internalizing antigen-containing ICs but compared with the activating FcγRIV under inflammatory conditions was found to elicit much weaker T cell activation, requiring a 30-fold antigen excess to reach comparable levels (36).

In addition to the priming of T cells which requires the antigen to be processed for presentation on MHC molecules, mouse B cells, *via* the B cell receptor (BCR), recognize antigen in the unprocessed state. This process can be facilitated by FcγRIIB which can recycle the antigen in an intact conformation permissive for recognition by the BCR (61). Such a mechanism has been suggested to be of particular importance in the context of humoral immunity involving T cell-independent B cell responses.

### FcRn Directed Shuttling of Endocytosed Antigen

**Figure 2:**
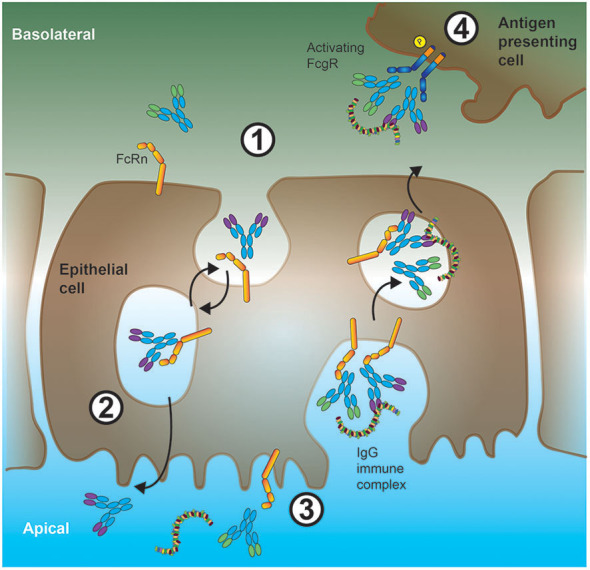
Interplay between FcRn and low-affinity FcγRs in shuttling antigen-ICs across epithelial barriers. 1. In the intestine, IgG antibodies can bind to FcRn expressed at the basolateral cell membrane of epithelial cells. These antibodies will then be internalized and shuttled bound to FcRn with high affinity inside the acidic microenvironment of endosomes. 2. On the apical (gut lumen oriented) side of the epithelial cell, due to the more neutral pH and consequently, reduced affinity of FcRn for IgG, these IgG antibodies are released, and can subsequently bind to soluble protein antigen. Alternatively (orange arrow), FcRn can shuttle monomeric IgGs back to the tissue interstitium (IgG recycling), a process preventing IgG degradation in sour compartments especially in APCs. 3. Luminal IgG-ICs can be internalized and shuttled, *via* endosomal vesicles, by binding to FcRn, through the epithelial cell soma. 4. These IgG-ICs are then released at the basolateral cell surface to the tissue interstitium, where they bind to low-affinity FcγRs on the cell surface of APCs.

The naming of the neonatal Fc receptor (FcRn) comes from its role in the transfer of IgG from mother to offspring across the intestinal epithelium in rats (62, 63) and the placenta in humans (64). However, expression of FcRn is not restricted to neonates. In fact, in adults, FcRn plays a crucial role in the maintenance of IgG levels and transport of IgG across epithelial barriers (65, 66) as illustrated in Figure 2. At the intestinal epithelial barrier, human FcRn can transport IgG into to the lumen to bind cognate antigen, and recycle the IgG-IC back across the intestinal barrier for subsequent uptake by mucosal DCs (67).

FcRn is predominantly detected in the endosomal compartment and constitutively cycles between there and the plasma membrane. At the low pH of these intracellular compartments, it binds to IgG with high affinity (62, 68, 69). Internalized monomeric IgG binding to FcRn in these acidic endosomal compartments and can be recycled to the cell surface where the IgG is released upon neutral pH change, thereby avoiding lysosomal destruction inside the cell and extending the half-life of IgG molecules in the circulation (70, 71).

In human APCs such as macrophages and DCs, FcRn is co-expressed with other FcγRs (72). As FcRn is predominantly intracellular and can bind IgG at acidic pH, it is ideally situated to engage IgG-ICs within endolysosomal compartments and to regulate IgG-IC trafficking and subsequently MHC class II-mediated antigen presentation (73–78). *In vitro* priming of mouse CD4^+^ T cells with ICs occurs far more efficiently with DCs from wild-type mice compared to those isolated from FcRn knockouts (75). The importance of FcRn in antigen presentation has been further confirmed by the use of IHH-IgG. These IgG molecules contain Fc mutations at three critical sites, I253A, H310A, and H435A, which retain FcγR binding but abrogate FcRn binding. ICs generated using IHH-IgG fail to efficiently stimulate CD4^+^ T cells compared to those generated with wt-IgG (75). FcRn has also been demonstrated to facilitate antigen cross-presentation by the CD8^−^CD11b^+^ DC subset (73). Nevertheless, it should be noted that in some settings, the IC enhancement of a response to antigen can proceed independently of FcRn as suggested in this mouse study (79).

At epithelial barriers, FcRn can transport IgG from the basolateral side to the apical lumen. A well-characterized example is the transport of IgG into the intestinal epithelium (67, 80). Critically, using a human FcRn model system, once the transported IgG engages its antigen, Yoshida et al. demonstrated transport back of the IgG-containing IC across the intestinal barrier where it was taken up by DCs in the *lamina propria* to facilitate antigen presentation (67) as summarized in Figure 2.

This could be important from a clinical perspective where intravenous immunoglobulin (IVIg) is commonly used in the treatment of autoimmune and inflammatory diseases (81). Through saturation of FcRn, IVIg increases clearance of pathogenic antibodies and may therefore reduce FcRn-mediated antigen-presentation as shown for example in autoimmune skin blistering diseases (82).

### The Molecular Machinery Downstream of FcγR

**Figure 3:**
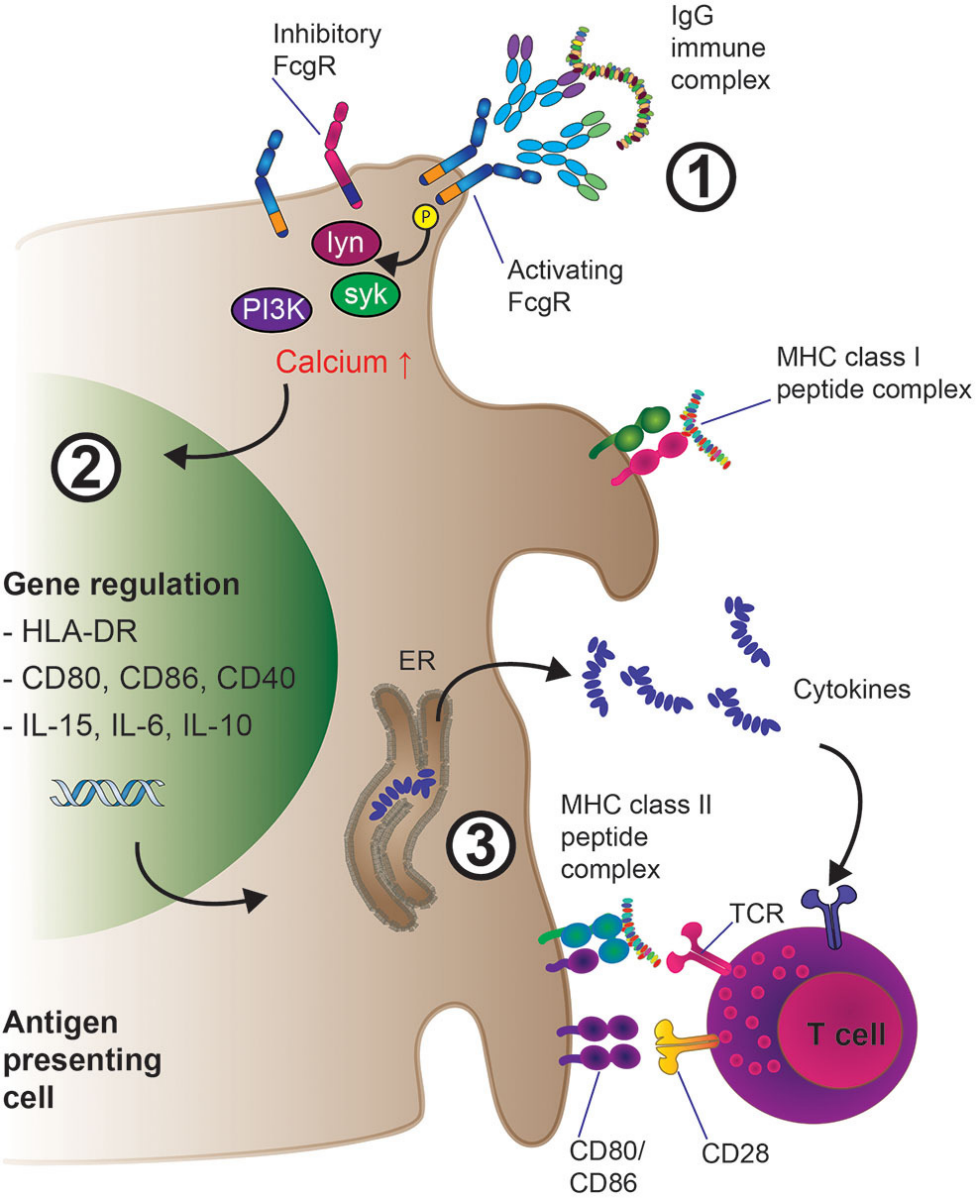
Mechanisms of enhanced antigen presentation and T cell activation downstream of IgG-IG binding to low-affinity FcγRs on APCs. 1. IgG-IC mediated cross-linking of activating low-affinity FcγRs leads to src kinase mediated ITAM phosphorylation, which induces the activation of several downstream cell-type specific kinases such as lyn, syk, and eventually PI3K, resulting in increased intracellular calcium and activation of the cell. Inhibitory (ITIM) containing FcγRs such as FcγRIIB can also be expressed at the cell surface; preferentially engaging inhibitory FcγR signaling would inhibit cellular activation as described in section the molecular machinery downstream of FcγR. 2. Cellular activation triggers transcriptional programs inside the APC such as enhanced expression of pro-inflammatory cytokines (e.g., IL-6, IL-15, IL-10) as well as up-regulation of membrane-bound co-activating molecules including CD80 and CD86. 3. IgG-IC mediated FcγR crosslinking results in MHC class I and II driven presentation of antigen-derived peptides to T cells. Subsequent T cell activation *via* T cell receptor: MHC interaction is enhanced by concomitant co-stimulation of the T cell through CD80/CD86 engagement of CD28 as well as priming of the immune response (i.e., Th1 vs. Th17) through pro-inflammatory cytokines released by the APC in the microenvironment.

The extracellular portion of FcγRs consist of two or more immunoglobulin (Ig)-like domains arranged in tandem, which interact with the CH (constant region, heavy chain) domains of IgG Fcs (31, 83). For signal transduction, FcγRs rely on tyrosine motifs in their intracellular portion, which in the case of activating FcγRs (human: FcγRI, FcγRIIA, FcγRIIIA; mouse: FcγRI, FcγRIII, FcγRIV) are termed immunoreceptor tyrosine-based activation motif (ITAM) (84). FcγRIIA contains an intrinsic ITAM motif, whilst FcγRIIIA and FcγRI associate with another polypeptide, the Fc receptor gamma chain (FcRγ). The Fc receptor gamma chain was initially described in the context of Fcε receptor signaling (85–88) and also contains an ITAM. FcRγ is not only important for activating cell signaling downstream of FcγRI and FcγRIII engagement (and FcγRIV in mouse), but also for the assembly and cell surface expression of the receptors (89). FcγR ITAM sequences are phosphorylated by scr kinases upon crosslinking leading to metabolic pathway activation and functional cell responses. Both cell type and FcγR determine which src kinase is activated. For instance, FcγRIIIA aggregation leads to lck activation in NK cells, while FcγRIIA or FcγRIIIA activate lyn and hck in monocytes and mast cells (90, 91). Subsequently, downstream of src kinase ITAM phosphorylation, src homology 2 (SH2) containing signaling molecules that bind the phosphorylated ITAM, most notably the Spleen tyrosine kinase (syk) kinase family, are recruited to the ITAM domains as illustrated in Figure 3. These events then lead to the activation of Phosphoinositide 3-kinase (PI3K) which in turn leads to production of Phosphatidylinositol [3–5]-trisphosphate (PIP_3_) and recruitment of Pleckstrin homology (PH) domain containing molecules, such as phospholipase C gamma (PLCγ) and Tyrosine-protein kinase (Tec). Myeloid cells contain Tec kinases such as Bruton's tyrosine kinase (Btk) and Itk among others, which can be activated upon FcγR crosslinking. In addition, in macrophages, SH2 domain containing leukocyte protein of 76kDa (SLP-76) and B-cell linker (BLNK) link Syk activation with Btk and PLCγ responses. Importantly, PLCγ activity leads to the generation of second messenger molecules: cytoplasmic soluble inositol trisphosphate (IP_3_) and cytoplasmic membrane bound diacylglycerol (DAG), which ultimately trigger cytoplasmic calcium mobilization and protein kinase C activation, respectively (92, 93). Depending on the cell type and context, FcγRI, FcγRIIA, and FcγRIIIA aggregation can lead to degranulation, phagocytosis, antibody-dependent cell-mediated cytotoxicity (ADCC), but also cytokine production and secretion of inflammatory mediators. Some of these activating FcγR mediated effects will be discussed later in this article.

Importantly, in the case of FcγRIIB, the inhibitory receptor in both mouse and human, signal transduction is not mediated *via* an ITAM domain, but *via* a related immunoreceptor tyrosine-based inhibition motif (ITIM) (94, 95). Here, FcγRIIB crosslinking also leads to src phosphorylation of the ITIM tyrosine residues, which is followed by signaling suppressing phosphatases of the SHIP or SHP-I and SHP-II class. Importantly, since inhibitory and activating FcγRs are often co-expressed on the cell surface, it is the ratio of these molecules and the strength of the IC:FcγR interaction which ultimately determines if a cell is activated or repressed by ICs, a phenomenon reviewed elsewhere (96). With regards to the therapeutic exploitation of this pathway, it was demonstrated that IVIg exerts its anti-inflammatory activity through the inhibitory FcγRIIB receptor (97). At least *in vitro*, IVIg has also been shown to inhibit MHC class II-dependent presentation of ovalbumin, albeit in this case in an FcγR independent manner (98).

More recently, an intracellular IgG receptor has been described, particularly in the context of antiviral immunity: namely, tripartite motif-containing 21 (TRIM21) (99). TRIM21 resides within the cytosol and recognizes the Fc-portion of IgG with high affinity. Binding of TRIM21 to antibody coated viral ICs not only results in proteosomal targeting and destruction of virions (100), but also enhanced DC maturation and cross-presentation of viral peptides to CD8 T cells (101).

In addition to mounting pro-inflammatory signaling and T cell activation downstream of IC challenge, human monocyte-derived macrophages co-incubated with high concentrations of ICs and LPS surprisingly assumed an anti-inflammatory phenotype (102). This resulted in enhanced tissue remodeling and release of angiogenetic factors, results which were confirmed in biopsies from patients with leprosy characterized by hypergammaglobulinemia and defective cell-mediated immunity. These findings suggest that macrophages may respond to IC challenge in a highly tissue and context-dependent fashion in the presence of FcgR cross-linking.

## FcγR Mediated Effects on APCs: Antigen Uptake and T Cell Priming

### FcγR and Immune Cell Function: Evidence From Knockout Mice

Efficient APC mediated antigen presentation to T cells is a crucial step in the generation of antigen specific adaptive immune responses. In the case of IC uptake, both the high-affinity FcγRI, as well as the activating low-affinity FcγRs, have been shown to be of critical importance. Mechanistically, the contribution of the different FcγRs has been convincingly shown using genetic knockout (KO) mouse models.

The first definitive genetic proof that activating FcγRs contribute to IC mediated activation of DCs and subsequent MHC class II, as well as MHC class I mediated antigen presentation, was obtained by using knockout (KO) mouse models deficient for the FcR common gamma chain, FcRγ (59). Here, the induction of DC maturation and the promotion of efficient MHC class I–restricted presentation of peptides from exogenous, IgG-complexed antigens was entirely dependent on functional FcRγ alleles. First, an FcRγ chain competent mouse DC cell line (D1), which expressed FcγRI, FcγRIIB, and FcγRIII, was challenged with ovalbumin:hen egg lysozyme IC (OVA:HEL-ICs). This effectively led to DC maturation and increase in MHC class II cell surface expression. Importantly, OVA specific CD8^+^ T cell hybrid cells (B3Z) were activated by the IC challenged DCs *in vitro* in a TAP dependent manner, suggesting effective cross presentation subsequent to IC internalization and cell activation. Using bone marrow (BM) derived primary DCs, it was further confirmed that activating FcγRs are indeed required for the IC mediated DC activation and antigen presentation by repeating the above series of *in vitro* experiments using FcRγ KO animals (89). Compared to wt control DCs, while the KO cells could still be activated by LPS in a similar manner, OVA-ICs failed to activate KO DCs, probably due to inefficient IC internalization.

While Regnault et al. showed that activating FcγRs critically contributed to IC uptake, other studies followed up using more candidate specific KO systems. Using a constitutive FcγRIII KO mouse model, human, or sheep erythrocytes opsonized with mouse mIgG1, mIgG2a or mIgG2b were incubated with macrophages and compared to wt macrophages (103). Whereas, IgG2a and IgG2b opsonized erythrocytes were efficiently phagocytosed by both wt and mutant macrophages, IgG1 opsonized erythrocytes were much less efficiently lysed. This indicated that IgG1 preferentially bound to FcγRIII expressing macrophages. Similarly, IgG1 containing, but not IgG2a or IgG2b ICs were much less efficiently trapped by follicular dendritic cells in spleens and lymph nodes of mutant animals, suggesting that this activating FcγRs are important in the internalization of IgG1 coated ICs.

In another mouse KO study, the contribution of the inhibitory FcγRIIB to IC uptake was investigated. Here, FcγRIIB KO BM derived DCs and macrophages were challenged with OVA-ICs and subsequent T cell activation was compared to wt or FcRγ deficient systems which exclusively express functional FcγRIIB (104). Interestingly, FcγRIIB negative DCs led to a reduced T cell activation, whereas FcRγ deficient but FcγRIIB expressing systems led to an increase in T cell activation. This suggested that not only activating FcγRs contributed to IC mediated T cell activation, but also the inhibitory FcγRIIB, which is the only FcγR expressed on B cells.

In a more recent *in vivo* study, OVA specific mouse strains selectively deficient for FcγRI/FcγRII/FcγRIII, FcγRI/III, or FcγRII only were challenged with polyclonal rabbit OVA-ICs, and subsequent CD8 OVA specific T cell responses were assessed (105). Importantly, simultaneous FcγRI/FcγRIII KO did not significantly impair OVA-specific CD8 T cell proliferation after *in vivo* challenge thus at first glance recapitulating the findings of Yada et al. However, in mice selectively lacking the inhibitory mouse FcγRIIB, there was no significant change in OVA-specific T cell activation compared to wt animals, suggesting that *in vivo*, all activating as well as the inhibitory FcγRIIB could contribute to IC uptake and APC activation followed by MHC class I antigen mediated CD8 T cell activation. Consequently, several FcγRs may compensate for individual KO indicating that there is no crucial single FcγR critical for IC mediated T cell activation.

The seemingly contradictory findings of Regnault et al. and Yada/de Jong et al. may be explained by the experimental conditions used in their studies. For instance, Regnault et al. focused primarily on DC biology. They employed *in vitro* or *ex vivo* assays using OVA as an antigen in the presence of ICs comprised of hen egg lysozyme (HEL) incubated with anti-HEL antibodies. These consisted of three monoclonal mouse anti-HEL antibodies (59, 106). Yada et al., however, employed OVA (and HEL) ICs containing rabbit IgG (104). Consequently, one explanation could be that differential FcγRIIB and FcγRIIIA affinities for mouse vs. rabbit IgG may explain these findings. In addition, de Jong et al. performed extensive *in vivo* studies. It is conceivable that the *in vivo* environment may be intrinsically different to the *in vitro* cell culture settings used by Regnault and Yada, especially considering the use of OVA-ICs consisting of non-host IgG in immunocompetent mice.

More recently, Lehmann et al. specifically investigated the role of different FcγRs on mouse splenic DC subsets (36). It was found that all subtypes of FcγRs (FcγRI, FcγRIIB, FcγRIII, and the mouse specific FcγRIV) were expressed on conventional mouse splenic DCs (cDCs). Using OVA-ICs, the authors then investigated which DC subset (and other FcγR expressing APCs) most efficiently internalized OVA-IC *via* a fluorescent quenching system allowing the distinction of internalized from total OVA-ICs. Interestingly, CD11c^+^CD8^+^ DCs were more efficient in the uptake of FcγR targeting antibodies than CD11c^+^CD8^−^ DCs. In contrast to monocytes, B cells (FcγRIIB^+^) did not internalize any OVA-ICs in this experiment; the authors suggested that this might be due to the expression of a non-endocytic FcγRIIB variant. NK and T cells did not internalize OVA-ICs, either. OVA-IC internalizing DCs then induced a short-lived T cell proliferation, which only became long-lasting if a broad adjuvant such as anti-CD40 and polyinosinic:polycytidylic acid (pIC) or heat-killed Listeria monocytogenes (HKLM) was co-applied. CD4^+^ T cell responses were best initiated *via* CD11c^+^CD8^−^ DCs, while CD11c^+^CD8^+^ DCs induced CD8^+^ T cell proliferation. T cell proliferation could be induced independently of the subtype of FcγR *via* which the OVA-IC uptake occurred (including the activating FcγRIV as well as the inhibitory FcγRIIB).

By using a mouse with intact FcγR expression at the DC surface but dysfunctional FcγR ITAM-mediated downstream signaling [the NOTAM model (107)], T cell activation downstream of IC challenge was assessed. ITAM signaling was found to be essential in mediating effector functions such as bone marrow-derived macrophage mediated ADCC (107). Nevertheless, in the NOTAM mouse, uptake of OVA-ICs by bone marrow-derived DCs was reduced (108). Consistent with the findings of Lehmann et al. (36), MHC II antigen presentation by splenic DCs was effective in NOTAM mice, although cross-presentation was abrogated (108). Other regulators of FcγR signaling, such as PTPN22, a susceptibility gene for multiple autoimmune diseases, may also regulate the antigen-presentation capability of DCs from mice (109). Bone marrow DCs from PTPN22 KO mice exhibit an enhanced capability to stimulate T cells with IC-derived antigens.

Taken together, these findings suggest that both activating and inhibitory FcγRs have the potential to regulate the capability of APCs to activate antigen-specific T cells.

### Additional Mechanisms of FcγR Enhancement of Antigen-Presentation

Upon FcγR crosslinking and antigen internalization, several important changes occur in APCs such as DCs or macrophages (110) which are illustrated in Figure 3. These include the activation and differentiation of the APC, which is accompanied by phenotypical changes, processing of the antigen (as discussed above) and the presentation of antigen-derived peptides *via* MHC class I and II molecules to T cells. T cell activation is triggered upon TCR interaction with MHC class I or MHC class II presented antigen peptide; in the course of antigen specific T cell activation, this first event is generally termed “signal 1.” However, signal 1 on its own is not sufficient to effectively activate T cells; in addition, cell-cell interaction *via* cell surface receptors and ligands (co-stimulatory proteins, termed “signal 2”) is required to enhance the duration of successful TCR:MHC interaction. Furthermore, cytokines present in the tissue microenvironment and secreted by the APC (“signal 3”) enhance the transcriptional downstream effects of signals 1 and 2 even more and help induce specific T helper cell responses broadly classified by the resulting T cell phenotype (Th1—pro-inflammatory, Th2—anti-inflammatory) or by the lead cytokine secreted by the Th population, [e.g., Th17 for IL-17 secreting CD4^+^ T cells (111, 112)]. In this respect, in recent years, it has become clear that FcγR crosslinking through ICs dramatically enhances an APC's ability to induce all components of effective T cell stimulation: MHC-II cell surface density (signal 1); the expression of cell surface bound co-stimulatory molecules (signal 2) as well as the release of pro- or anti-inflammatory cytokines (signal 3). In this section, we will discuss crucial insights into these processes.

Firstly, FcγR crosslinking can result in increases in cell surface co-stimulatory molecules. Murine DCs incubated OVA-IC upregulate not only MHC II molecules, but also the co-stimulatory molecule CD86 which can co-stimulate T cells *via* CD28 (59). In the same study, it was shown that CD40 is also induced at the cell surface of APCs; the CD40: CD154 axis (the CD40 ligand expressed on activated T cells) has also been shown to play an important role in T cell activation (113). These changes were monitored by flow cytometry and occurred within 24 h post-challenge; intriguingly, at least within this time frame, OVA alone (the “naked” antigen) was not able to induce these cell surface expression level changes, highlighting the importance of FcγR crosslinking in the efficient activation of APCs.

In addition to providing T cells with co-stimulatory signals *via* cell surface molecules, FcγR engagement can also trigger the release of soluble factors such as cytokines that can increase or skew T cell activation. In a mouse study on bone marrow (BM) derived DCs challenged with mouse IgG-ICs or unchallenged cells, subsequent gene expression analysis by microarray furthermore corroborated the finding that ICs strongly induce T cell polarizing cytokine regulation (114). The authors also included a KO mouse model deficient in FcRγ. Importantly, the cytokine genes *IL2, IL6, IL10, IL15, IL23a, IL27*, and *Ifnb1* were upregulated in WT BM-DCs, but not FcRγ chain KO BM-DCs after incubation with ICs, highlighting that the intracellular signaling cascade initiated by ITAM phosphorylation, an early event following FcγR crosslinking, has long-lasting transcriptional changes in APC biology.

Dendritic cell phagocytosis of IgG-opsonized bacteria *via* FcγRIIA in conjugation with TLR stimulation enhanced DC production of IL-1β and IL-23. These cytokines promote differentiation of Th17 cells and accordingly T cell responses were skewed toward a Th17 response. Other lines of evidence suggest a cross-talk between TLRs and FcγRs driving cytokine production. Exposure of human macrophages to IgG-ICs in conjunction with TLR ligands amplifies production of TNF-α, IL-1β, and IL-6, all of which play critical roles in the pathology of diseases such as rheumatoid arthritis (RA) (115). Important differences in FcγR-triggered cytokine production between humans and mice have been reviewed by Vogelpoel et al. (116). Finally, our own studies using human primary cell-derived assay systems showed that stronger T cell responses to tetanus toxoid were induced by human monocyte derived DCs, or in total PBMC cultures, when challenged with the IgG-coated antigen as opposed to the “naked” antigen alone (117, 118). Tetanus toxoid IgG-ICs significantly enhanced the production of pro-inflammatory cytokines IFN-γ, TNF-α, and IL-1β, whilst FcγR-blockade with a multivalent Fc-containing molecule, or with a cocktail of FcγR blocking antibodies, abrogated this response confirming the importance of FcγRs in mediating this enhancement.

Taken together, these findings suggest that antigen uptake, processing, and presentation, depending on the tissue microenvironment, lead to the increased expression of MHC-II and co-stimulatory cell surface molecules such as CD86 and CD40 in APCs. In addition, transcriptional up-regulation and subsequent production and release of pro-inflammatory cytokines in both mouse and human is triggered upon FcγR crosslinking and antigen internalization, which further enhance T cell polarization including pro-inflammatory Th1 and Th17 T cell responses. Furthermore, the findings in this section clearly suggests that FcγRs through IgG-ICs drive a much more pronounced and distinct type of APC activation compared to “naked” antigen uptake alone further highlighting the biological importance of these receptors.

### FcγR Variants in Health and Disease: Evidence From Genetic Variation

Assorted genetic variants of FcγRs have been shown to impact the affinity for and interaction with different IgG isotypes (40), a finding which has been associated with disease and clinical potential of therapeutic antibodies (119–121). In the clinic, polymorphisms often affect FcγR function by interfering with their affinity for IgG antibodies, or their trafficking and cellular localization, which in turn has been proposed to contribute to the pathogenesis of IC driven autoimmune diseases (122) exemplified by systemic lupus erythematosus (SLE).

SLE is a chronic autoimmune disease characterized by tissue IC deposition (123) in a variety of organs including the skin and kidney (lupus nephritis), which often leads to severe clinical complications (123). In SLE, genetic predisposition has been established, and susceptibility genes linked to SLE include FcγR loci including FcγRII and FcγRIII on chromosome 1q23 (124, 125). In addition, in the case of inhibitory FcγRIIB, the hypothesis that receptor dysfunction may indeed be linked to SLE was initially suggested by a mouse KO model in which loss of FcγRIIB resulted in spontaneous development of murine SLE (126). Since then, human studies have investigated in more detail genetic polymorphisms in SLE patients.

In the case of the aforementioned inhibitory FcγRIIB, a single amino acid polymorphism, FcγRIIBT232 was initially discovered in a Japanese SLE patient population (127). This polymorphism, 695T > C, resulted in a non-synonymous substitution, Ile232Thr (I232T), within the transmembrane domain of FcγRIIB. The authors further showed that FcγRIIBT232 was in some cases linked with polymorphisms affecting activating FcγRIIIA. This hypothesis was experimentally tested by generating humanized mice with normal and functionally impaired FcγRIIB including FcγRIIBT232 (128). Humanized mice with this mutation produced higher levels of serum IgM and IgG, and although all B cells in the peripheral blood expressed FcγRIIB on their cell surface they were shown to be functionally impaired. Notably, these mice began to produce autoantibodies directed against double-stranded DNA, glucose phosphate 6-isomerase, rheumatoid factor, and made antibodies directed against cyclic citrullinated peptides, typical for SLE patients. Together, these findings suggested that in SLE, FcγRIIBT232 may result in failure to inhibit activating low-affinity FcγRs, which in turn may promote a general pro-inflammatory and exaggerated humoral response to ICs.

Importantly, in a subsequent study on 1296 healthy white control subjects, only 1% of the donors were homozygous for aFcγRIIbT232, strengthening both its disease association as well as highlighting possible differences between ethnically different SLE patient subsets (129). In the same study, the cell biological effects of FcγRIIBT232 were studied in detail (129). Using a monocytic cell line, U937, which normally only expresses activating FcγRI and FcγRIIA, the authors showed that activation of the cells was inhibited if they were transfected with wt FcγRIIB. When challenged with FcγRIIA crosslinking antibodies, U937 wt cells internalized and shuttled the cargo to cathepsin D positive lysosomes, whereas the FcγRIIB transfectants showed reduced intracellular shuttling. Of note, if U937 cells were transfected with the FcγRIIBT232 variant as opposed to wt FcγRIIB, there was no reduction in intracellular cargo shuttling. Similarly, *via* antibody mediated receptor aggregation, FcγRI mediated superoxide generation was reduced in wt, but not FcγRIIBT232 transfected cells. These results were confirmed studying the phagocytosis of opsonized *Streptococcus pneumonia* bacteria. This strongly suggested that the FcγRIIBT232 polymorphism leads to functional impairment of FcγRIIB resulting in the inability to dampen activating FcγR function. Compared to its wt counterpart, FcγRIIBT232 resulted in paradoxical de-phosphorylation, altered phosphatase interaction, and phosphorylation independent recruitment of SHIP. Also, FcγRI triggered phospholipase D (PLD) activity, which is associated with lipid rafts (130), was inhibited by expression of FcγRIIB (wt) but not FcγRIIB1T232. Since both FcγRI and FcγRIIB depend on lipid rafts, membrane separation experiments were carried out showing that only wt FcγRIIB, but not FcγRIIBT232, was readily detectable in the lipid raft fraction of U937 cells. Conclusively, these studies show that lack of inhibitory FcγRIIB function, either by complete loss of function or due to genetic polymorphisms, result in increased activating FcγR signaling in response to ICs, thus generating an immunological environment favoring SLE.

In addition to FcγRIIB, the activating FcγRIIA and FcγRIII have also been studied in the context of SLE (131). For FcγRIIA, two co-dominantly expressed versions have been identified, R131 and H131, with the 131-Arg (R131) allele binding IgG2 much less avidity than the 131-His (H131) allele (132). Only the homozygous expression of the R/R131 variant has been associated with increased susceptibility of SLE and earlier onset (133, 134). For FcγRIII, sequencing studies suggested that a variant, FcγRIIIA-176F, which displays lower binding of IgG1 and IgG3 compared to its polymorphic variant, FcγRIIIA-176V, could be considered an SLE risk allele (133, 135).

Together, in addition to the role of polymorphisms in FcγRIIB, these studies suggest that polymorphisms in activating low affinity FcγRs may also contribute to SLE susceptibility, potentially *via* a shift in affinity for different IgG isotypes and which may consequently entail different immunological responses to ICs, (e.g., in lupus nephritis). However, genetic susceptibility seems to be linked to different ethnicities in SLE patient populations, and the mechanistic and cell biological effects of the genetic variants remain less well-understood.

## Summary

The FcγR family provides a critical point of control for regulating antigen presentation of IgG-coated antigens. This control can be exerted at several stages such as the internalization of antigen, directing intracellular antigen transport, and by controlling subsequent signaling events that lead to the presentation of antigen. Stimulatory or inhibitory signals generated by FcγRs lead to regulation of co-stimulatory molecules and cytokines that further modulate the response to an antigen. The importance of these pathways to human disease is highlighted by the growing number of Fc gamma receptor genetic variants linked to human biology.

## Author Contributions

FJ, JG, and OQ conceptualized, wrote, and reviewed the manuscript. FJ made the article illustrations. All authors contributed to the article and approved the submitted version.

## Conflict of Interest

This work was conceived out of personal dedication as a joint effort between FJ, OQ, and JG and was not funded by their employers. FJ is currently an employee of F. Hoffmann-La Roche Ltd, and a former employee of UCB Pharma. OQ is an employee of Celentyx Ltd and a former employee of UCB Pharma. JG is currently an owner and Director of Celentyx Ltd.

## Abbreviations

aa: Amino acids
ADCC: Antibody-dependent cell-mediated cytotoxicity
APC: Antigen presenting cell
BCR: B cell receptor
BLNK: B-cell linker
BM: Bone marrow
Btk: Bruton's tyrosine kinase
CD: Cluster of differentiation
cDC1: myeloid/conventional DC1
cDC2: myeloid/conventional DC2
CH: Constant region, heavy chain
CTL: Cytotoxic T lymphocyte
DAG: Diacylglycerol
DC: Dendritic cell
ELISA: Enzyme-linked immunosorbent assay
ER: Endoplasmic reticulum
Fc: Fragment, crystallizable
FcRn: Neonatal Fc receptor
FcRγ: Fc receptor common gamma chain
FcγR: Fc gamma receptor
HEL: Henn-egg lysozyme
HKLM: Heat-killed Listeria monocytogenes
HLA: Human leukocyte antigen
IC: Immune complex
IgG: Immunoglobulin type G
IL: Interleukin
IP_3_: Inositol trisphosphate
ITAM: Immunoreceptor tyrosine based activation motif
ITIM: Immunoreceptor tyrosine based inhibitory motif
IVIg: Intravenous immunoglobulin
KLH: Keyhole limpet hemocyanin
KO: Knockout
lck: Lymphocyte-specific protein tyrosine kinase
LPS: Lipopolysaccharide
lyn: Lck/Yes novel tyrosine kinase
MHC: Major histocompatibility complex
NK: Natural killer cell
OVA: Ovalbumin
pDC: Plasmacytoid dendritic cell
PBMC: Peripheral blood mononuclear cell
PH: Pleckstrin homology domain
PI3K: Phosphoinositide 3-kinase
pIC: Polyinosinic:polycytidylic acid
PIP_3_: Phosphatidylinositol [3–5]-trisphosphate
PLC: Phospholipase C
PLD: Phospholipase D
PPD: Purified protein derivative
SH2: Src Homology 2
SLE: Systemic lupus erythematosus
SLP-76: SH2 domain containing leukocyte protein of 76kDa
Src: Sarcoma (kinase)
Syk: Spleen tyrosine kinase
TAP: Transporter associated with antigen presentation
TCR: T cell receptor
Tec: Tyrosine-protein kinase
TH: T helper cell
TLR: Toll-like receptor
TNF: Tumor necrosis factor
TRIM21: Tripartite motif-containing 21
wt: Wildtype.
